# An intersectional expression platform for gene complementation using RNA-fragment end joining (REJ)

**DOI:** 10.64898/2026.09.13.751279

**Published:** 2026-09-14

**Authors:** Lukas C. Bachmann, Ryan H. Hsu, Kip J. Hermann, Claire E. Williams, Richard L. Farman, Nicolas Criales, Craig Clark, Stella Kramer, Carolina Thörn Perez, Sawyer Randles, Karen Lettieri, Samuel L. Pfaff

## Abstract

Gene complementation is a powerful tool for genetic selection and protein functional studies but typically requires extensive screening for complementary components. Here we engineered a platform for gene complementation based on RNA end-joining (REJ) that precisely and efficiently splices separate RNA units into functional coding mRNAs. REJ is mediated by short structured modular RNA segments of ∼250bp that: (a) promote RNA::RNA interaction, (b) coopt the intrinsic cell splicing machinery to facilitate RNA trans-splicing, (c) minimizes translation of protein fragments from un-spliced RNA segments, and (d) encodes scar-free protein. We demonstrate that REJ is a broadly applicable system that can reliably split nearly any gene into complementary segments regardless of protein structure. To enable the use of this system we provide a toolbox of reporters and a web-based design tool to facilitate vector design. Demonstrated REJ applications include genetic complementation, intersectional labeling of cell types, and efficient expression of large proteins.

**Highlights:**

- Synthetic RNA cis-elements can direct efficient and precise RNA trans-splicing *in vivo* .
- Endogenous splicing factors catalyze RNA end-joining (REJ) without foreign proteins.
- The REJ platform can be applied to nearly any gene with few positional constraints.
- REJ can be used as an intersectional tool to build complex genetic logic circuits.

## Full Text Availability

The license terms selected by the author(s) for this preprint version do not permit archiving in PMC. The full text is available from the preprint server.

